# Geographic variation in dengue seroprevalence and force of infection in the urban paediatric population of Indonesia

**DOI:** 10.1371/journal.pntd.0006932

**Published:** 2018-11-02

**Authors:** Clarence C. Tam, Megan O’Driscoll, Anne-Frieda Taurel, Joshua Nealon, Sri Rezeki Hadinegoro

**Affiliations:** 1 Saw Swee Hock School of Public Health, National University of Singapore and National University Health System, Singapore, Singapore; 2 Faculty of Epidemiology and Population Health, London School of Hygiene & Tropical Medicine, London, United Kingdom; 3 Department of Infectious Disease Epidemiology, Imperial College London, London, United Kingdom; 4 Sanofi Pasteur, Asia and JPAC Region, Singapore, Singapore; 5 Department of Child Health, Faculty of Medicine, Universitas Indonesia, Cipto Mangunkusumo Hospital, Jakarta, Indonesia; University of Heidelberg, GERMANY

## Abstract

Understanding the heterogeneous nature of dengue transmission is important for prioritizing and guiding the implementation of prevention strategies. However, passive surveillance data in endemic countries are rarely adequately informative. We analyzed data from a cluster-sample, cross-sectional seroprevalence study in 1–18 year-olds to investigate geographic differences in dengue seroprevalence and force of infection in Indonesia. We used catalytic models to estimate the force of infection in each of the 30 randomly selected sub-districts. Based on these estimates, we determined the proportion of sub-districts expected to reach seroprevalence levels of 50%, 70% and 90% by year of age. We used population averaged generalized estimating equation models to investigate individual- and cluster-level determinants of dengue seropositivity. Dengue force of infection varied substantially across Indonesia, ranging from 4.3% to 30.0% between sub-districts. By age nine, 60% of sub-districts are expected to have a seroprevalence ≥70%, rising to 83% by age 11. Higher odds of seropositivity were associated with higher population density (OR = 1.54 per 10-fold rise in population density, 95% CI: 1.03–2.32) and with City (relative to Regency) administrative status (OR = 1.92, 95% CI: 1.32–2.79). Our findings highlight the substantial variation in dengue endemicity within Indonesia and the importance of understanding spatial heterogeneity in dengue transmission intensity for optimal dengue prevention strategies including future implementation of dengue vaccination programmes.

## Author summary

Understanding the geographic distribution of dengue transmission intensity is of key importance for guiding dengue prevention strategies, including vaccination. We analyzed age-stratified data from a cross-sectional survey of 30 randomly selected urban sub-districts in Indonesia and estimated the force of infection (FOI) in each. Substantial variation in FOI estimates were observed, ranging from 4% to 30% between sub-districts. Heterogeneity which will be important to understand when considering future vaccine introduction in Indonesia. Higher odds of dengue seropositivity were associated with increasing levels of urbanization, which may represent areas where more people could benefit from dengue vaccination or should otherwise be prioritized for dengue control.

## Introduction

Dengue is the most widely distributed mosquito-borne viral infection; 40% of the world’s population is at risk, three-quarters of whom live in the Asia-Pacific region [1–3]. However, the burden of dengue disease remains poorly quantified in many dengue endemic countries in Asia because existing passive surveillance systems capture only a small fraction of all dengue cases, often relying on clinical diagnoses which excludes milder and atypical presentations of disease [4,5].

Indonesia is one of the largest countries in the dengue endemic region, with a population of 260 million, more than half of whom live in urban areas. Dengue transmission in Indonesia is hyper-endemic, with co-circulation of all four dengue serotypes. In 2013, the Ministry of Health of Indonesia reported 112,511 cases of dengue (41.3 per 100,000 population) and 871 deaths, corresponding to a case fatality rate of 0.7% [6]. Variable application of surveillance case definitions, health-seeking behaviour and lack of laboratory confirmation means that the rates of dengue infection and disease are likely to be heavily underestimated [7,8]. In a longitudinal study of dengue burden in high-incidence populations within five Southeast Asian countries (Indonesia, Malaysia, Thailand, the Philippines and Vietnam), the rate of virologically-confirmed dengue in healthy Indonesian children aged 2–14 years was 3.6 cases per 100 person-years, more than 10 times that detected by national surveillance data. The sensitivity of clinical diagnosis in this research environment in Indonesia was 59% [9,10]. Of the five countries, the Indonesian cohort experienced the highest rate of virologically-confirmed dengue hospitalizations (1.6 hospitalizations per 100 person-years) and dengue haemorrhagic fever (0.6 episodes per 100 person-years) [9].

Dengue transmission can exhibit significant temporal and geographical variability even at small spatial scales, with large variations in dengue incidence sometimes observed in neighbouring administrative units [11,12]. Drivers of such differences in dengue transmission may be multifactorial, with climatic variables, level of urbanization, socioeconomic factors and vector ecology likely to be playing significant roles. Determining the roles of these factors in local dengue transmission can help inform decisions about where prevention and control strategies may be most needed.

In September 2016, Indonesia approved Dengvaxia (Sanofi Pasteur), a live-attenuated, chimeric, tetravalent dengue vaccine. The vaccine is recommended for use in individuals who have already experienced dengue infection [13]. The World Health Organization (WHO) Strategic Advisory Group of Experts on Immunization (SAGE) previously issued guidelines for implementation of the vaccine based on local transmission intensity, recommending countries consider introducing the vaccine according to seroprevalence thresholds of approximately 70% or greater in the age group targeted for vaccination [14]. These age-related serological thresholds remain important to consider when assessing population-level impacts and the cost-effectiveness of dengue vaccination strategies [14–16].

Knowledge of sub-national dengue transmission intensity levels can be extremely valuable to policy makers when considering the geographic regions and population age-groups that may benefit most from prevention and control interventions. Only one nationwide, age-stratified dengue seroprevalence survey has been conducted in Indonesia, in the 2014 urban paediatric population, the results of which are reported by Prayitno et al. [17]. In this paper, we use the geographically-stratified design of this survey to describe the variation in dengue seroprevalence and force of infection across Indonesia, with a view to inform public health policy decisions surrounding dengue vaccination and other prevention strategies.

## Methods

### Ethics statement

Ethical approval for the original survey was obtained from the Health Research Ethics Committee of Faculty of Medicine of University of Indonesia. Signed, informed consent was obtained from a parent or legal guardian of each child for participation in the study as well as from participants aged 13–18 years. In addition, signed assent was obtained from participants aged 8–12 years old. Additional approval for this secondary analysis was obtained from the Ethical Review Committee of the London School of Hygiene and Tropical Medicine.

### Seroprevalence survey

A cross-sectional seroprevalence survey, nationally-representative of the urban population, was carried out between 30^th^ October and 27^th^ November 2014. The cluster sampling design was self-weighted and adapted from the WHO Expanded Program on Immunization (EPI) cluster survey method [18]. The survey design and characteristics of the survey population have been described in detail by Prayitno et al. [17]. Briefly, 30 urban sub-districts in Indonesia were randomly selected for the study based on probability proportional to population size. Only sub-districts with a minimum population of 1000 were included in the sampling list to ensure the desired sample size was obtained. Within each sub-district, 107 healthy children aged 1–18 years old were enrolled, stratified by age-group. The study aimed to enrol 660 children aged 1–4 years (22 per cluster), 870 aged 5–9 years (29 per cluster), 870 aged 10–14 years (29 per cluster) and 810 aged 15–18 years (27 per cluster), giving a total sample size of 3,210 children. Children were included in the study if they were aged 1–18 years on the day of enrolment, were reported as healthy by a parent or legal guardian, and had been resident in the selected study site for more than one year. Children were excluded if they had fever (axillary temperature ≥37.5°C) at the time of recruitment or on the day of blood sampling, if any other child from the household was already included in the study, or if they were unable to report to the local *puskesmas* (health centre) for blood drawing within 7 days of the household visit.

### Laboratory analysis

Each 2ml sample of venous blood was centrifuged, aliquoted and the serum frozen and stored at -20°C. All samples were transported to and processed at the Eijkman Institute for Molecular Biology in Jakarta, Indonesia. The dengue sero-status of each sample was determined using the commercial Panbio Dengue IgG enzyme-linked immunosorbent assay (ELISA) as per the manufacturer’s instructions (Panbio units <9 is negative; 9–11 is equivocal; and >11 is positive) [17].

### Data analysis

#### Force of infection

A catalytic model was used to estimate the national average force of infection, *λ*, assuming an age-independent seroconversion rate. This force of infection (FOI), *λ*, is the predicted rate at which individuals of age *a* move from a seronegative to a seropositive state, thereby giving an average rate of seroconversion per year of life [19]. The catalytic model predicts the proportion of children seropositive by age *a*, *p*_*a*_, using the following equation:

$$p_{a}=1-e^{-\lambda a}$$

We fitted an age-constant model to the serological data using a binomial regression model with a complementary log-log link as follows:

$$ln(-ln(1-p_{a}))=ln(\lambda )+ln(a)$$

The constant term in this model, *ln(λ)*, is the natural logarithm of the force of infection. The natural logarithm of age (the midpoint of each age category in years) is included as an offset, with its coefficient constrained to 1. We used the model to estimate a nationally representative FOI and associated 95% confidence intervals (95% CI), using robust standard errors to adjust for data dependency within survey clusters. We also estimated cluster-specific FOI. Based on these estimates, we calculated the proportion of clusters that reached a seroprevalence of 50%, 70% and 90% at each year of age.

Additionally, we investigated age-varying models that relaxed the assumption of a fixed seroconversion rate at all ages using linear piece-wise constants. The Wald test was used to compare model fit.

#### Individual and cluster-level determinants of dengue seropositivity

For each sub-district, information was obtained on the latitudinal and longitudinal coordinates of the approximate geographic centroid, as well as the population density as reported in the 2010 Indonesian census [20]. Region-level data were also obtained from the World Bank Indonesia Database for Policy and Economic Research on the proportion of households with safe water access, the household per capita expenditure (in IDR), the proportion of households with access to safe sanitation, the human development index (HDI) and the immunization coverage of children under five years [21]. These were all based on 2013 statistics, except for the human development index which was only available for 2010. We also used information on the region administrative status (City or Regency). This classification is based on demography, size and economy, with City status usually defined by having non-agricultural economic activities [22].

We used generalized estimating equation (GEE) models to investigate associations between dengue seropositivity and individual- and cluster-level variables [23]. The outcome variable for this analysis was individual dengue IgG antibody seropositivity. The model formulation was as follows:

$$logit\;P(Y_{ij}=1|X_{ij})=\alpha +\beta_{1}X_{1ij}+\beta_{2}X_{2ij}+\cdots +\beta_{k}X_{kij}$$

where *P(Y*_*ij*_ *= 1)* represents the probability that an individual *j* in survey cluster *i* is seropositive, *X*_*1ij*_ to *X*_*kij*_ represent values of 1 to *k* independent variables for individual *j* in survey cluster *i*, and *β*_*1*_ to *β*_*k*_ are the corresponding regression coefficients representing population-averaged effects. We included as individual-level regressors age, sex and self-reported number of dengue cases in the household since the participant's birth, as identified in the original paper by Prayitno *et al*. Age (in years) was fitted as a continuous, quadratic variable to allow for deviations from linearity. We also investigated potential associations between seropositivity and the above-mentioned district and sub-district level variables. Population density was fitted as a continuous variable on the logarithmic (base 10) scale, as initial analysis indicated that this provided a better fit to the data. The proportion of households with safe water access and household per capita expenditure were grouped into quintiles. We constructed a multivariable, population-averaged effects model using a forward stepwise approach, including variables in the model one at a time and assessing their effect on the outcome using odds ratios (OR) and 95% confidence intervals (95% CI) derived from robust standard errors, as well as Wald test p-values. We evaluated model fit using the Quasilikelihood Information Criterion (QIC), favouring models with a lower value of QIC [24]. We verified that the model was not sensitive to the model building procedure by repeating the analysis using a backward stepwise variable selection procedure. All analyses were conducted in Stata 14.0 (Stata Corporation).

## Results

**Fig 1:**
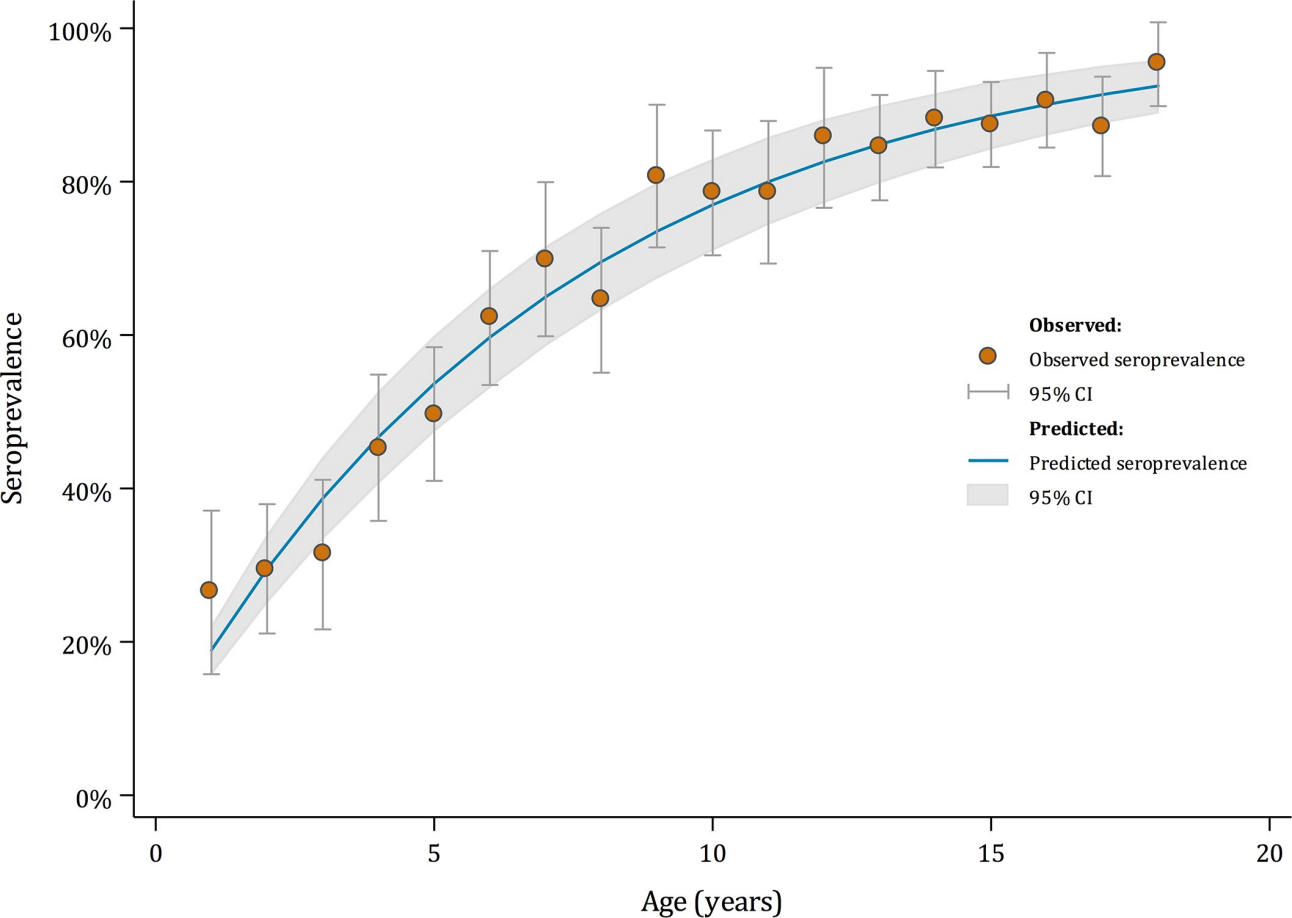
Observed (orange markers) and model-predicted (blue line) seroprevalence by year of age, based on an overall estimated force of infection, λ, of 14.0% per annum, with corresponding 95% confidence intervals, among children aged 1 to 18 years in Indonesia, 2014.

A total of 3,194 children were enrolled in the survey, of whom 2,216 (69%, 95% CI: 65–74%) tested positive for dengue IgG antibodies. Seroprevalence ranged from 26.5% (95% CI: 17.2–38.4%) amongst one year-olds to 95.3% (95% CI: 85.7–98.6%) amongst 18 year-olds. Assuming an age-constant FOI, we estimated that 14.0% (95% CI: 11.8–16.6%) of seronegative children in Indonesia experience their first infection each year, shown in Fig 1. Models allowing for age-varying forces of infection did not provide a better fit to the data than the age-constant model.

**Fig 2:**
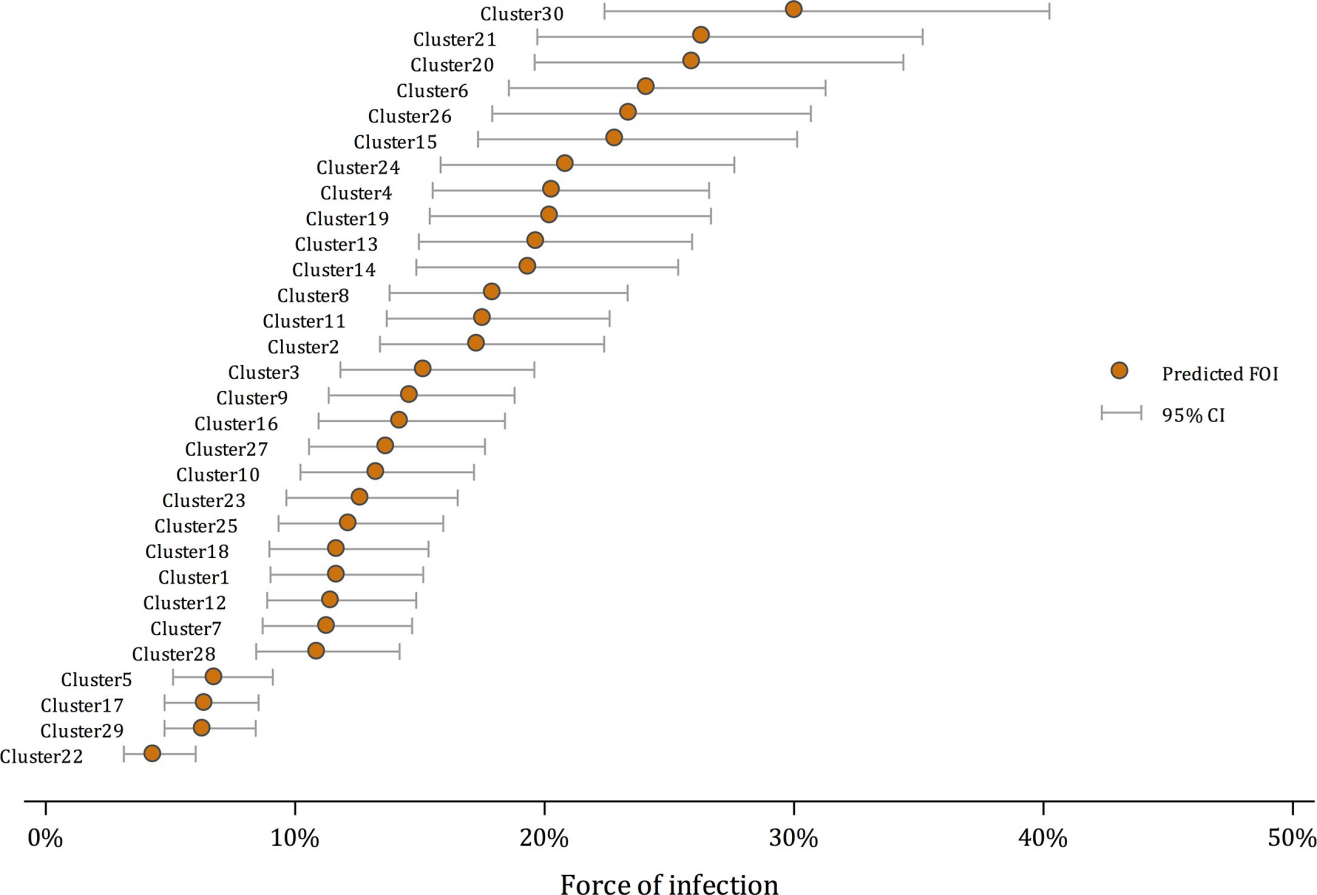
Estimated cluster-specific annual forces of infection with associated 95% confidence intervals among children aged 1 to 18 years in Indonesia, 2014.

**Fig 3:**
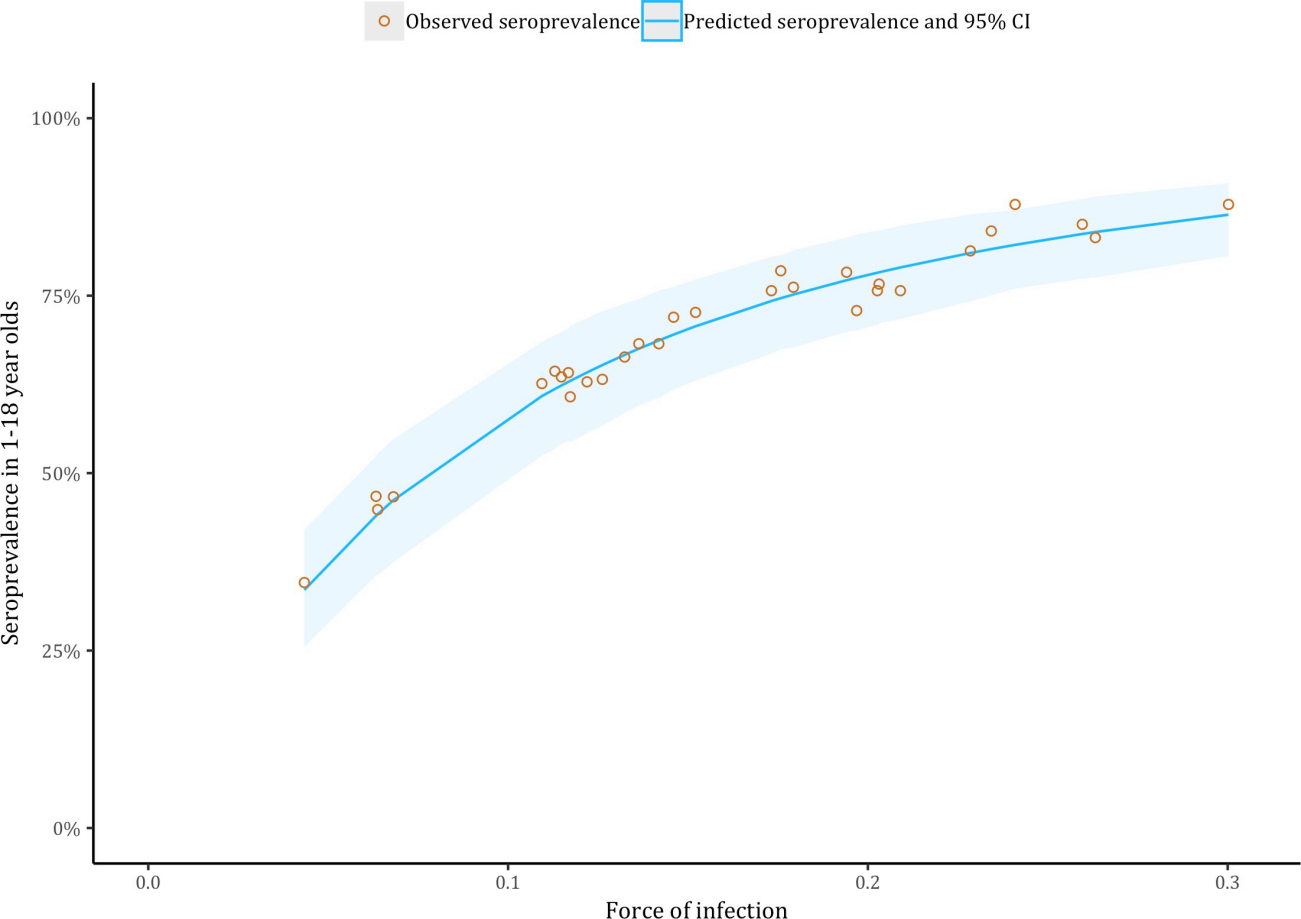
Observed (orange markers) and model-predicted (blue line) cluster-specific seroprevalence against estimated cluster-specific force of infection among children aged 1 to 18 years in Indonesia, 2014.

There was substantial variation in seroprevalence between clusters, ranging from 34.6% to 87.9%. Fig 2 shows the variation between clusters in the predicted FOI, ranging from 4.3% (95% CI: 3.1–6.0%) per year in cluster 22, to 30.0% (95% CI: 22.4–40.2%) per year in cluster 30, see S1 Table for full details. The relationship between force of infection estimates and the observed and predicted total seroprevalence amongst 1–18 year olds is shown in Fig 3, where predicted seroprevalence is calculated assuming an age-constant force of infection and a uniform age-distribution.

**Fig 4:**
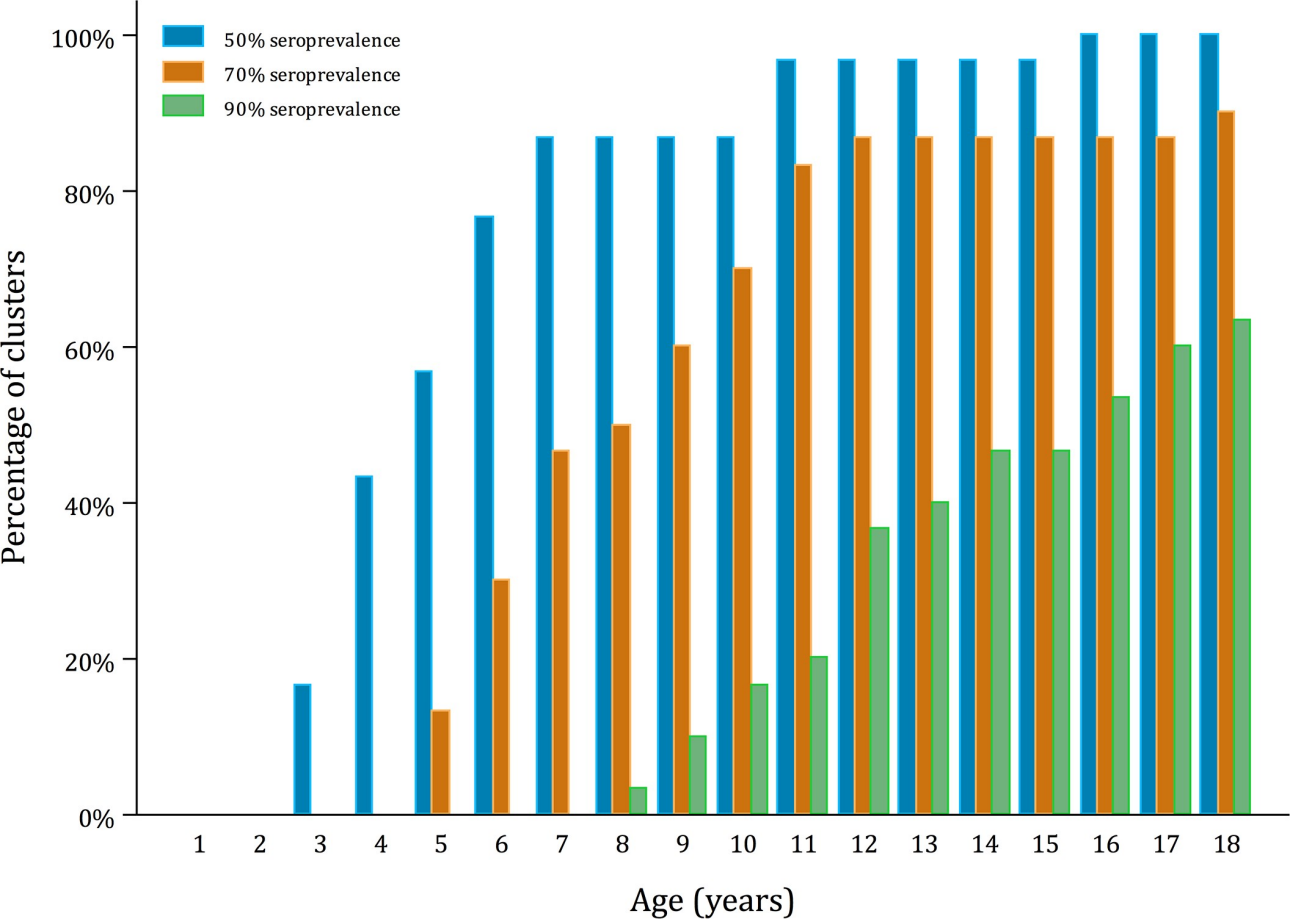
Proportion of clusters expected to reach seroprevalence levels of 50%, 70% and 90% by year of age, based on models with an age-constant force of infection.

Fig 4 shows the percentage of sub-districts that reach a seroprevalence of 50%, 70% and 90% by year of age, based on the predicted force of infection estimates. Our models show that by age nine 87% (26/30), 60% (18/30) and 10% (3/30) of sub-districts will have reached a seroprevalence level ≥50%, ≥70% and ≥90%, respectively. By age eleven 97% (29/30), 83% (25/30) and 20% (6/30) have respectively reached these levels of seroprevalence.

Initial assessment of correlations between independent variables indicated a strong correlation between district-level household per capita expenditure and the proportion of households with access to safe water (Spearman's rho = 0.77), and only the former was included in multivariable analyses. Human Development Index, immunization coverage and geographic coordinates showed no association with dengue seropositivity and were not included in multivariable analysis. In the final multivariable GEE model controlling for age, sex and the self-reported number of dengue cases in the household since the participant's birth, cluster-level variables associated with dengue seropositivity included district-level administrative status, household per capita expenditure, and sub-district level population density (Table 1). The odds of seropositivity were 92% higher among individuals residing in districts with City as opposed Regency administrative status (OR = 1.92, 95% CI: 1.32–2.80), and increased by 54% on average per 10-fold increase in population density (OR = 1.54, 95% CI: 1.03–1.71) between the range of 241–38,182 inhabitants per km^2^. The backward stepwise variable selection procedure yielded the same final model.

**Table 1:** Factors associated with dengue seropositivity in children aged 1–18 years, results from multivariable population-averaged effects model, Indonesia 2014.

		95% CI		
Variable	OR	Lower	Upper	p^4^
Age^1^				
*Linear age term*	1.53	1.37	1.71	<0.001
*Squared age term*	0.99	0.98	0.99	
Sex				
*Female*	1.00	—	—	0.048
*Male*	0.86	0.74	1.00	
Population density^2^				
*Per 10-fold increase*	1.54	1.03	1.71	0.037
Household per capita expenditure				
*1st quintile*	1.00	—	—	<0.001
*2nd quintile*	1.64	0.76	3.54	
*3rd quintile*	2.79	1.25	6.23	
*4th quintile*	1.39	0.65	2.95	
*5th quintile*	0.77	0.37	1.59	
Administrative status				
*Regency*	1.00	—	—	0.006
*City*	1.92	1.32	2.80	
Household dengue cases^3^				
*0*	1.00	—	—	<0.001
*1*	2.38	1.62	3.50	
*>1*	3.78	1.28	11.14	
*Don't know*	1.28	0.93	1.74	
*No data*	1.07	0.56	2.06	

^1^Age modelled as a continuous, quadratic term

^2^Population density modelled on logarithmic (base 10) scale, the minimum and maximum values were 241 and 38,182 inhabitants per km^2^

^3^Self-reported dengue cases in household since participant's birth

^4^Wald test p-value

## Discussion

To our knowledge, this is the first detailed analysis of within-country variation in dengue seroprevalence and force of transmission. Financial and logistical challenges in conducting large-scale, geographically widespread serological studies mean that such surveys are often restricted to a very limited number of sites. Our data, from a cluster-sample survey representative of urban areas in Indonesia, provided a unique opportunity to study geographic variation in dengue transmission. Our results indicate a high dengue transmission intensity in the paediatric population of Indonesia, with 14.0% seroconverting per year, similar to estimates found in Colombo, Sri Lanka (14.1%) in children aged 0–12 years [14]. The survey was not designed to estimate force of infection at sub-national level and further work, for example supplementing seroprevalence information with sub-national dengue notification data, could help to determine how representative the estimates are of different administrative levels. However, our analyses highlight the highly heterogeneous nature of dengue transmission in Indonesia, with sub-district seroprevalence ranging from 35% to 88%. These findings are relevant for informing dengue vaccination strategies, as they highlight the within-country variation in force of infection and therefore the variability in the expected proportion of seropositive individuals in any one age group targeted for vaccination. Importantly, based on the predicted FOI estimates, 60% of clusters reached a seroprevalence of ≥70% by age nine years, rising to 83% of clusters by age 11 years.

We found higher population density and City status to be associated with elevated odds of dengue seropositivity. This is not surprising given the epidemiology of dengue virus, which is primarily transmitted by the mainly urban *Aedes aegypti* mosquito. Higher population density is likely to be an indicator of increasing urbanization and higher transmission risk.

A number of limitations should be considered when interpreting our analysis. The original survey was designed to be nationally representative rather than provide estimates of seroprevalence by geographic region. Consequently, the sample size in each cluster was modest and the self-weighted survey design meant that clusters were sampled favourably from areas with higher population size. As a result, more sparsely populated areas were under-represented in our analysis. The survey was also confined to urban areas. Nevertheless, the availability of data from 30 locations presented a unique opportunity to investigate geographic variation in dengue transmission.

We assumed a constant force of infection over time in our analyses. Whilst our age-varying model for the total study population showed no evidence of variation in FOI by age, past epidemics could have resulted in specific age groups having disproportionately high seroconversion rates, potentially inflating force of infection estimates, particularly when analysing cluster-specific data. However, this is unlikely to explain our observation that certain clusters had considerably lower seroprevalence and force of infection than others.

The clustered, age-stratified sampling design enabled assessment of the relationship between cluster-level seroprevalence and force of infection in 1–18 year olds. Our catalytic models estimating cluster-level force of infection fit the data well and could largely reproduce the total seroprevalence in individual clusters assuming a uniform age-distribution (Fig 3). As the sampling strategy of individual clusters was approximately evenly distributed by age-group (21% 1–4, 27% 5–9, 27% 10–14, and 25% 15–19) we expect these results to be largely generalizable to similarly age-stratified surveys in paediatric populations. Force of infection estimates from age-constant catalytic models are invaluable for estimating the long-term average infection rates required for assessment of vaccine suitability.

In our survey, dengue seropositivity was determined using a commercial IgG ELISA. IgG seropositivity measures past exposure to at least one dengue serotype, but provides no information about exposure to individual serotypes, precluding estimation of serotype-specific forces of infection. However, force of infection estimates from IgG seroprevalence data have been shown to be comparable to the sum of serotype-specific forces of infection derived from plaque reduction neutralization tests (PRNT) [25]. A further complication of IgG ELISA results is the possibility of cross-reactivity with antibodies against other flaviviruses, including those raised following vaccination against Japanese encephalitis and yellow fever viruses. Previous studies in US travellers have shown high levels of false positive results when using manufacturer recommended diagnostic thresholds, suggesting a need to optimise cut-offs by validating ELISA tests against PRNT results [26]. We expect this to be much less of a problem in Indonesia, where dengue virus is the predominant circulating flavivirus and where there was no Japanese encephalitis immunization programme at the time of the survey [27,28]. Indeed, PRNTs in a third of IgG positive samples from our survey indicated that 98% were positive for at least one dengue serotype [29].

Our analysis of geographic determinants of seroprevalence was limited by the availability of data with adequate geographic resolution. Data on drinking water infrastructure, vector densities and weather factors were not available at the sub-district level and therefore not accounted for in this analysis. Further research to establish the role of climate and other determinants in dengue transmission across Indonesia could help to predict areas or periods of high dengue transmission intensity, in order to effectively target control measures or further characterize geographic regions that could benefit from a dengue vaccination campaign.

This study highlights the value of geographically-stratified dengue seroprevalence data to understand the variation in dengue epidemiology, and inform prevention and control strategies, including dengue vaccination. Our study describes the variability in dengue endemicity which is observed between individual sub-districts within urban areas of a dengue-endemic country. Largely urbanized areas with high population densities may be where implementation of dengue control and prevention strategies would be of most value.

## Supporting information

S1 Table Estimated force of infection and 95% confidence intervals by cluster. (DOCX)

S1 File Description of supporting data files. (DOCX)

S2 File Cluster-level variables. (CSV)

S3 File Dengue IgG serology results by survey cluster and year of age. (CSV)
