# Supplementary material for: Geographic variation in dengue seroprevalence and force of infection in the urban paediatric population of Indonesia
Source: PLoS Negl Trop Dis. 2018 Nov 2;12(11):e0006932. doi: 10.1371/journal.pntd.0006932 (PMC6241133; doi:10.1371/journal.pntd.0006932)
Supplement: S1 Table — (DOCX) [file pntd.0006932.s001.docx]

| Cluster ID | N | Province | Regency | Sub-district | FOI (95%CI) |
| --- | --- | --- | --- | --- | --- |
| 1 | 106 | NANGGROE ACEH DARUSSALAM | SUBULUSSALAM | SIMPANG KIRI | 0.12 (0.09-0.15) |
| 2 | 107 | SUMATERA UTARA | MEDAN | MEDAN DENAI | 0.17 (0.13-0.22) |
| 3 | 106 | SUMATERA BARAT | PADANG | PAUH | 0.15 (0.12-0.20) |
| 4 | 107 | JAMBI | BUNGO | BUNGO DANI | 0.20 (0.16-0.27) |
| 5 | 105 | LAMPUNG | LAMPUNG SELATAN | KALIANDA | 0.07 (0.05-0.09) |
| 6 | 107 | BANTEN | TANGERANG | CIKUPA | 0.24 (0.19-0.31) |
| 7 | 101 | BANTEN | TANGERANG | BENDA | 0.11 (0.09-0.15) |
| 8 | 105 | DKI JAKARTA | JAKARTA SELATAN | PESANGGRAHAN | 0.18 (0.14-0.23) |
| 9 | 107 | DKI JAKARTA | JAKARTA TIMUR | PULO GADUNG | 0.15 (0.11-0.19) |
| 10 | 107 | DKI JAKARTA | JAKARTA BARAT | KALI DERES | 0.13 (0.10-0.17) |
| 11 | 107 | JAWA BARAT | BOGOR | GUNUNG PUTRI | 0.18 (0.14-0.23) |
| 12 | 107 | JAWA BARAT | BANDUNG | BANJARAN | 0.11 (0.09-0.15) |
| 13 | 107 | JAWA BARAT | CIREBON | GUNUNG SARI | 0.20 (0.15-0.26) |
| 14 | 106 | JAWA BARAT | BEKASI | CIKARANG UTARA | 0.19 (0.15-0.25) |
| 15 | 107 | JAWA BARAT | BANDUNG | BOJONGLOA KALER | 0.23 (0.17-0.30) |
| 16 | 107 | JAWA BARAT | BEKASI | BEKASI TIMUR | 0.14 (0.11-0.18) |
| 17 | 107 | JAWA BARAT | TASIKMALAYA | SINGAPARNA | 0.06 (0.05-0.09) |
| 18 | 107 | JAWA TENGAH | KLATEN | TRUCUK | 0.12 (0.09-0.15) |
| 19 | 107 | JAWA TENGAH | JEPARA | PECANGAAN | 0.20 (0.15-0.27) |
| 20 | 107 | JAWA TENGAH | TEGAL | DUKUHTURI | 0.26 (0.20-0.34) |
| 21 | 107 | JAWA TENGAH | TEGAL | TEGAL BARAT | 0.26 (0.20-0.35) |
| 22 | 107 | JAWA TIMUR | PONOROGO | PULUNG | 0.04 (0.03-0.06) |
| 23 | 106 | JAWA TIMUR | BANYUWANGI | CLURING | 0.13 (0.10-0.17) |
| 24 | 107 | JAWA TIMUR | MOJOKERTO | NGORO | 0.21 (0.16-0.28) |
| 25 | 105 | JAWA TIMUR | SUMENEP | KALIANGET | 0.12 (0.09-0.16) |
| 26 | 107 | JAWA TIMUR | SURABAYA | SAWAHAN | 0.23 (0.18-0.31) |
| 27 | 107 | BALI | DENPASAR | DENPASAR SELATAN | 0.14 (0.11-0.18) |
| 28 | 107 | KALIMANTAN TIMUR | SAMARINDA | SAMARINDA ULU | 0.11 (0.08-0.14) |
| 29 | 107 | SULAWESI SELATAN | TORAJA UTARA | RANTEPAO | 0.06 (0.05-0.08) |
| 30 | 107 | SULAWESI TENGGARA | KENDARI | KENDARI | 0.30 (0.22-0.40) |

Supplementary Table 1. Cluster/Sub-district force of infection estimates and 95% confidence intervals.
