# Supplementary material for: Geographic variation in dengue seroprevalence and force of infection in the urban paediatric population of Indonesia
Source: PLoS Negl Trop Dis. 2018 Nov 2;12(11):e0006932. doi: 10.1371/journal.pntd.0006932 (PMC6241133; doi:10.1371/journal.pntd.0006932)
Supplement: S1 File — (DOCX) [file pntd.0006932.s002.docx]

# Geographic variation in dengue seroprevalence and force of infection in the urban paediatric population of Indonesia

## Supplementary data files

| **File** | **Description** |
| --- | --- |
| cluster_data.csv | contains district level indicators |
| cluster_sero.csv | contains number of dengue positive and negative individuals by cluster and year of age |

## Variable descriptions

file: cluster_data.csv

| **Variable** | **Description** |
| --- | --- |
| cluster_id | ID of survey cluster |
| regency | Regency of survey cluster |
| status | Regency/City status |
| popden | Population density (inhabitants / km^2^) |
| sanitation | 2013 household access to safe sanitation (% of total households) |
| safewater | Household access to safe water (% of total households) |
| hh_expend | 2013 household per capita expenditure (in IDR) |
| hdi | 2011 Human Development Index |
| vax_cover | 2013 immunisation coverage for children under 5 years old (% of population under 5 years old) |

## Variable descriptions

file: cluster_sero.csv

| **Variable** | **Description** |
| --- | --- |
| cluster_id | ID of survey cluster |
| age | Age (in single years) |
| neg | Number dengue IgG negative |
| pos | Number dengue IgG positive |
| total | Total participants |
